# Vertical structure and occurrence patterns of the cross-equatorial northerly surge under different ENSO and MJO phases

**DOI:** 10.1038/s41598-024-80951-9

**Published:** 2024-11-24

**Authors:** Qoosaku Moteki

**Affiliations:** Japan Agency for Marine-Earth Science and Technology (JAMSTEC), 2-15 Natsushima-Cho, Yokosuka City, 237-0061 Kanagawa Japan

**Keywords:** Atmospheric dynamics, Climate-change impacts

## Abstract

This study investigated the vertical structure of 6 cross-equatorial northerly surge (CENS) events during the Year of Maritime Continent–Cold Surge Observation in 2021 (YMC-CSO2021) campaign. These events, named CENS1 (Jan. 18–20), CENS2 (Jan. 29–30), CENS3 (Feb. 2–5), CENS4 (Feb. 5–9), CENS5 (Feb. 18–20), and CENS6 (Feb. 25–26), occurred under different environmental conditions associated with cold surges (CSs) and the Madden–Julian Oscillation (MJO). Using radiosonde observations, we identified distinct characteristics in the northerly wind layer thickness, westerly wind bursts, and potential temperature anomalies among the events. Notably, CENS6 featured a deep northerly wind layer reaching 400 hPa, influenced by a southward pressure gradient linked to a cyclone in the Southern Hemisphere. Statistical analysis of past CENS events using reanalysis dataset showed that 80% occurred in January and February, with higher frequencies during La Niña years and active MJO phases over the western Pacific, aligning with YMC-CSO2021 observations. Composite analysis showed that CENS events induced significant ascending motion and localized potential temperature gradients, leading to positive precipitation anomalies around the Maritime Continent. These findings enhance our understanding of CENS dynamics and their impact on regional climate variability.

**Supplementary Information:**

The online version contains supplementary material available at 10.1038/s41598-024-80951-9.

## Introduction

**Fig. 1:**
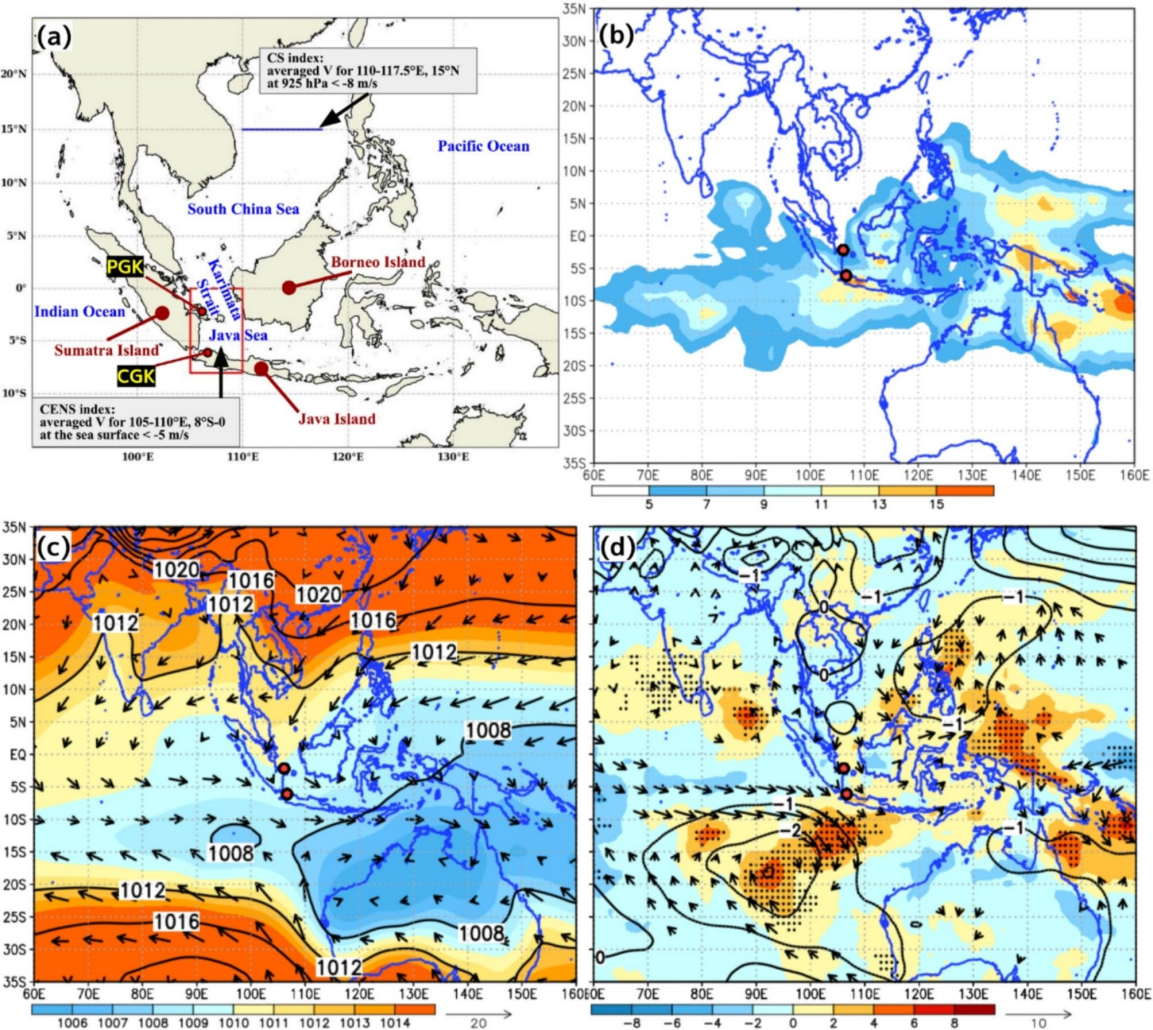
(**a**) Topographic map of the MC showing the regions relevant to this study. The calculation regions for CENS and CS indices are labeled. The two red circles indicate the locations of intensive radiosonde observations at CGK and PGK. Horizontal distribution depicting (**b**) precipitation with GPCP (color, mm/day), (**c**) SLP (color and contour, hPa), and 10-m wind vectors from JRA-55 and (**d**) precipitation anomalies (colors, mm/day), SLP anomalies (contours, hPa), and anomalous 10-m wind vectors averaged for the YMC-CSO2021 period from 8 January–8 March 2021. The anomalies are referenced to the climatological mean for December, January, February, and March 1997–2022. The results that are significant at the 95% confidence level are marked by black stipples and wind vectors in (**d**). The map in (**a**) was generated using Python 3.9.6 (http://www.python.org) including matplotlib 3.8.4 and cartopy 0.23.0. The plots in (b-d) were generated with GrADS v2.2.1 (http://cola.gmu.edu/grads/grads.php).

In the winter season in the Northern Hemisphere, the pressure gradient force between the high-pressure system over the Eurasian continent and the low-pressure system over the Australian continent drives southward winds across the Maritime Continent (MC)^1^. Cold air outbreaks, known as cold surges (CSs), originate from the southern Eurasian continent and extend into the South China Sea, significantly impacting regional precipitation on the MC^2,3^. Furthermore, the phenomenon in which northerly winds cross the equator from the southern South China Sea into the Karimata Strait and Java Sea is called cross-equatorial northerly surge (CENS). CENS is defined as strong northerly winds exceeding 5 m/s at the surface over the Karimata Strait and Java Sea^4^ (Fig. 1a).

The importance of studying CENS lies not only in its role in enhancing precipitation through interactions with diurnal convection, the Madden–Julian Oscillation (MJO)^5^, and tropical wave disturbances over the Sumatra and Java Islands, but also in its potential impact on the broader climate system of the region^6–11^. The variability in precipitation over the MC is intricately influenced by both east‒west traveling phenomena, such as the MJO and tropical wave disturbances, as well as variations in meridional wind components such as the CS and CENS. While there has been extensive research on the surface-level impact of CENS in enhancing precipitation over the MC region^4,6–10^, the understanding of the three-dimensional convergence and ascending motion associated with CENS remains insufficient. Since precipitation patterns in the MC region are influenced by various factors, analyzing the vertical structure of winds and temperature related to CENS is crucial for clarifying its relation with other factors.

During the Years of the Maritime Continent–Cold Surge Observation in 2021 (YMC-CSO2021)^12^ from January 8 to March 8, 2021, intensified radiosonde observations were conducted in collaboration with the Agency for Meteorology, Climatology, and Geophysics of the Republic of Indonesia (BMKG) and the Japan Agency for Marine-Earth Science and Technology (JAMSTEC). Observations were made at the Soekarno-Hatta International Airport (CGK, 6.12°S, 106.65°E) and Pangkal Pinang Airport (PGK, 2.17°S, 106.13°E) stations, which were strategically located to capture the southern and northern parts of the CENS, respectively (Fig. 1a). The 6 CENS events were observed under different environmental conditions associated with the MJO and CS variations. Although CENSs are defined as variations in surface northerly winds and their relationship with precipitation variability has been well studied^4,6–9^, their three-dimensional structure remains largely unexplored. This paper aims to examine the three-dimensional structures of the 6 CENS events observed during the YMC-CSO2021 and compares them with the statistical characteristics of past CENS events from 1959 to 2022, including monthly occurrence frequency, event duration, and their associations with ENSO and MJO phases. Through this analysis, this study aims to contribute to closing the gap in understanding the vertical structure of CENS and its implications for regional climate dynamics.

## Results

### Vertical structure of the 6 CENS events observed during the YMC-CSO2021

Figure 1 shows the horizontal distributions of precipitation, sea level pressure (SLP), and 10-m winds and their anomalies from the climatological mean averaged for the YMC-CSO2021 period from January 8 to March 8, 2021. During the YMC-CSO2021, northerly winds, as the basic state, were steadily dominant over the southern South China Sea, Karimata Strait, and Java Sea between the SLP ridge meridionally extending from the Indochina Peninsula and the low SLP area to northern Australia, and large precipitation areas were distributed in the vicinity of Java Island (Fig. 1b,c). The northerly winds around the CGK and PGK stations were significantly stronger than the climatological mean (Fig. 1d). In fact, an extreme precipitation event (> 200 mm/day) inducing widespread flooding occurred in association with the CENS event around the northern coast of Central Java Island on 5–6 February^7^. Hermawan et al. (2022) reported that this extreme precipitation event was closely linked to the CENS occurrence. In addition, positive precipitation and negative SLP anomalies were distributed over the eastern Indian Ocean and western Pacific Ocean and were associated with active convection of the MJO.

During the YMC-CSO2021 period, the following 6 events were extracted using the CENS index^4^ (see Fig. S1), as shown in Table 1. CENS1 (Jan. 18–20), CENS2 (Jan. 29–30), CENS3 (Feb. 2–5), CENS4 (Feb. 5–9), CENS5 (Feb. 18–20), and CENS6 (Feb. 25–26) each had different conditions for the CS index^3^ and the phase and amplitude of the MJO index^13^ (see Fig. S2). Among the observed 6 CENS events, CENS1, CENS2, CENS4, and CENS5 were associated with CS conditions, whereas CENS3 and CENS6 were not. In addition, CENS1, CENS2, CENS3, CENS4, CENS5, and CENS6 occurred during MJO phases 4, 6, 6, 7, 7, and 6, respectively. Specifically, CENS3, CENS4, CENS5, and CENS6 occurred during the strong amplitude of the MJO index > = 1 associated with active convection.

**Table 1:** Conditions of the 6 CENS events, indicating the presence or absence of a CS and the phase and amplitude of the MJO. “CS/nonCS” denotes whether a CS was present, with CS defined as an event in which the average 925-hPa northerly wind from JRA-55 between 110°E and 117.5°E along 15°N exceeded 8 m/s. “MJO status” specifies the MJO phase (1–8) and amplitude (active ≥ 1, inactive < 1).

	CENS1 (Jan. 18–20)	CENS2 (Jan. 29–30)	CENS3 (Feb. 2–5)	CENS4 (Feb. 5–9)	CENS5 (Feb. 18–20)	CENS6 (Feb. 25–26)
CS/nonCS	CS	CS	nonCS	CS	CS	nonCS
MJO status	Phase 4, inactive	Phase 6, inactive	Phase 6, active	Phase 7, active	Phase 7, active	Phase 6, active

**Fig. 2:**
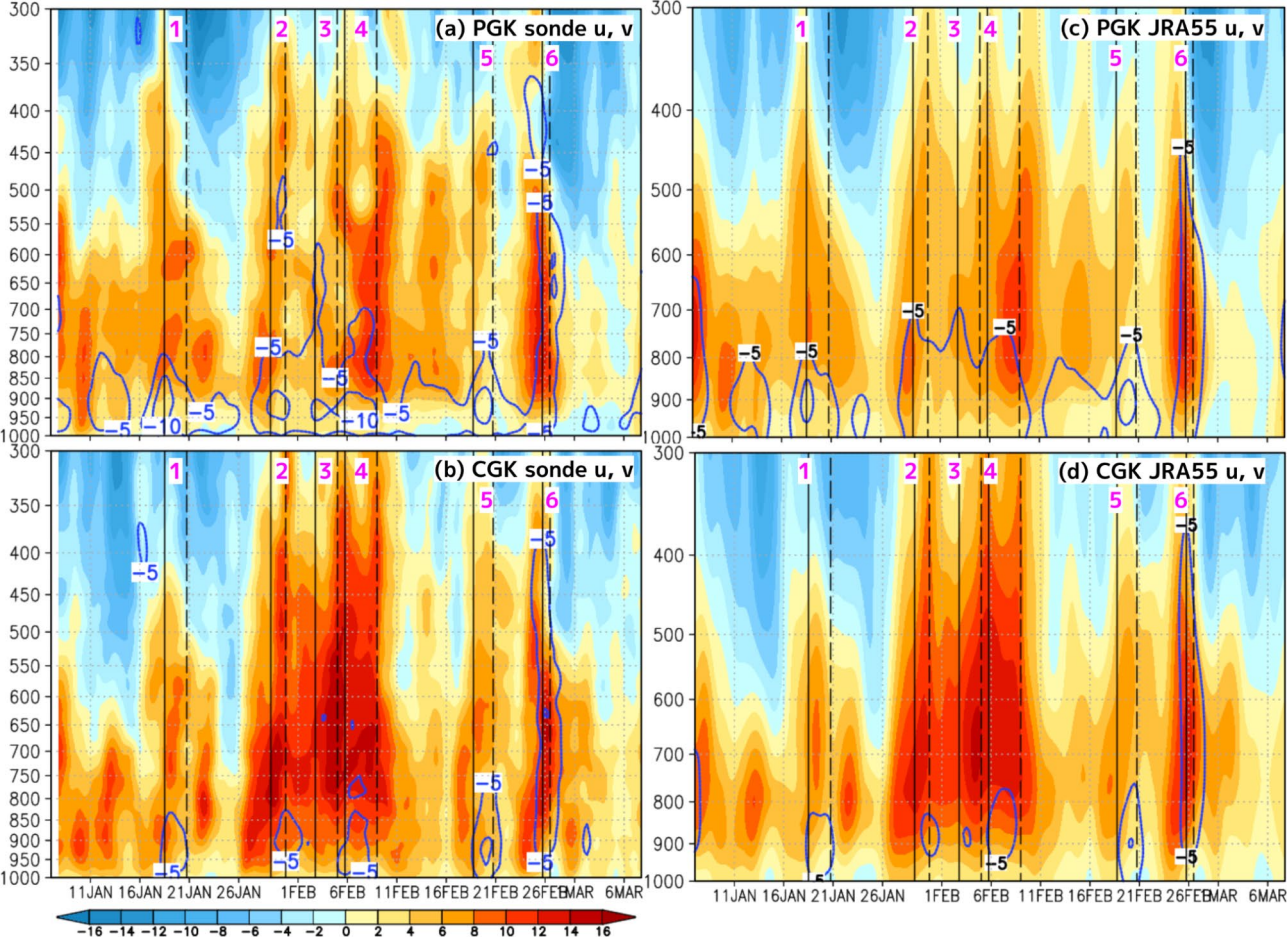
Time–height cross-sections of the zonal wind velocity (colors, m/s) and northerly wind velocity (blue contours every 5 m/s) from January 8 to March 8, 2021. (**a**,**b**) Represents radiosonde observations at PGK and CGK, respectively. (**c**,**d**) represents the JRA-55 reanalysis data for the PGK and CGK locations, respectively. A 24-hour moving average was applied to vertical profiles recorded four times per day to exclude diurnal variations. The solid and dashed lines indicate the onset and end dates of the 6 CENS events. The purple numbers denote the CENS events: CENS1, CENS2, CENS3, CENS4, CENS5, and CENS6. All plots were generated with GrADS v2.2.1 (http://cola.gmu.edu/grads/grads.php).

**Fig. 3:**
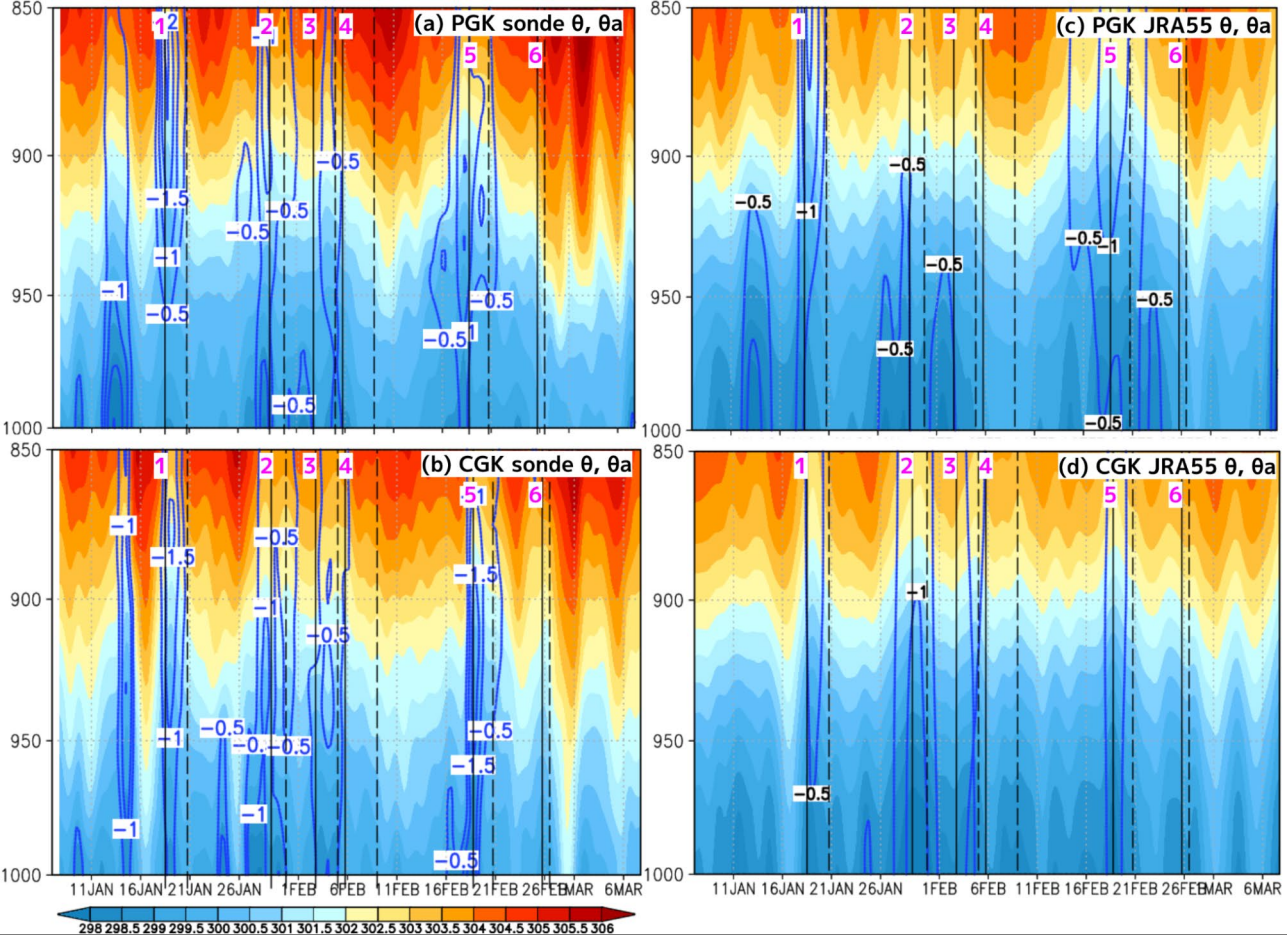
The same in Fig. 2 but for the PT (colors, K); the PT anomaly from the temporal mean for the entire YMC-CSO2021 period (blue contours every 0.5 K).

The vertical structure of winds in the 6 CENS events occurring under such different environmental conditions showed distinct characteristics in the radiosonde observations at PGK and CGK (Fig. 2a,b). At PGK, which was located in the northern part of the CENS, when examining the northerly wind layer thickness using a 5 m/s threshold, CENS1 and CENS5 were confined to the lower atmosphere from the surface up to 800 hPa, whereas the northerly winds associated with CENS2, CENS3, CENS4, and CENS6 extended above 800 hPa (Fig. 2a). At CGK, which was located in the southern part of the CENS, the northerly wind layer thickness for CENS1, CENS2, CENS3, CENS4, and CENS5 was limited to below 800 hPa from the surface, but for CENS6, it reached 400 hPa (Fig. 2b). Furthermore, when examining the layer of strong westerlies exceeding 8 m/s at CGK, the characteristics of the zonal winds varied significantly by event, especially for CENS2, CENS3, and CENS4, which extended to 300 hPa. This fact indicates that CENS2, CENS3, and CENS4 occurred under environmental conditions in which westerly wind bursts developed due to enhanced convective activity associated with the MJO over the western Pacific Ocean (corresponding to Phases 6–7 with the MJO index). The vertical structure of the potential temperature (PT) in the radiosonde observations at PGK and CGK revealed the thermal characteristics of the 6 CENS events (Fig. 3a,b). CENS1, CENS2, CENS3, and CENS5 were associated with cold anomalies of −0.5 K to −1 K, whereas CENS4 and CENS6 did not exhibit such cold anomalies.

By comparing the vertical profiles at the PGK and CGK locations from JRA-55 with the radiosonde data, it was possible to quantitatively and accurately represent both the differences and similarities in the vertical structures of the observed CENS northerly winds, westerly winds, and PT anomalies (Figs. 2c,d and 3c,d). This fact is very important as a premise for the statistical analysis with the JRA-55 shown in the next section.

**Fig. 4:**
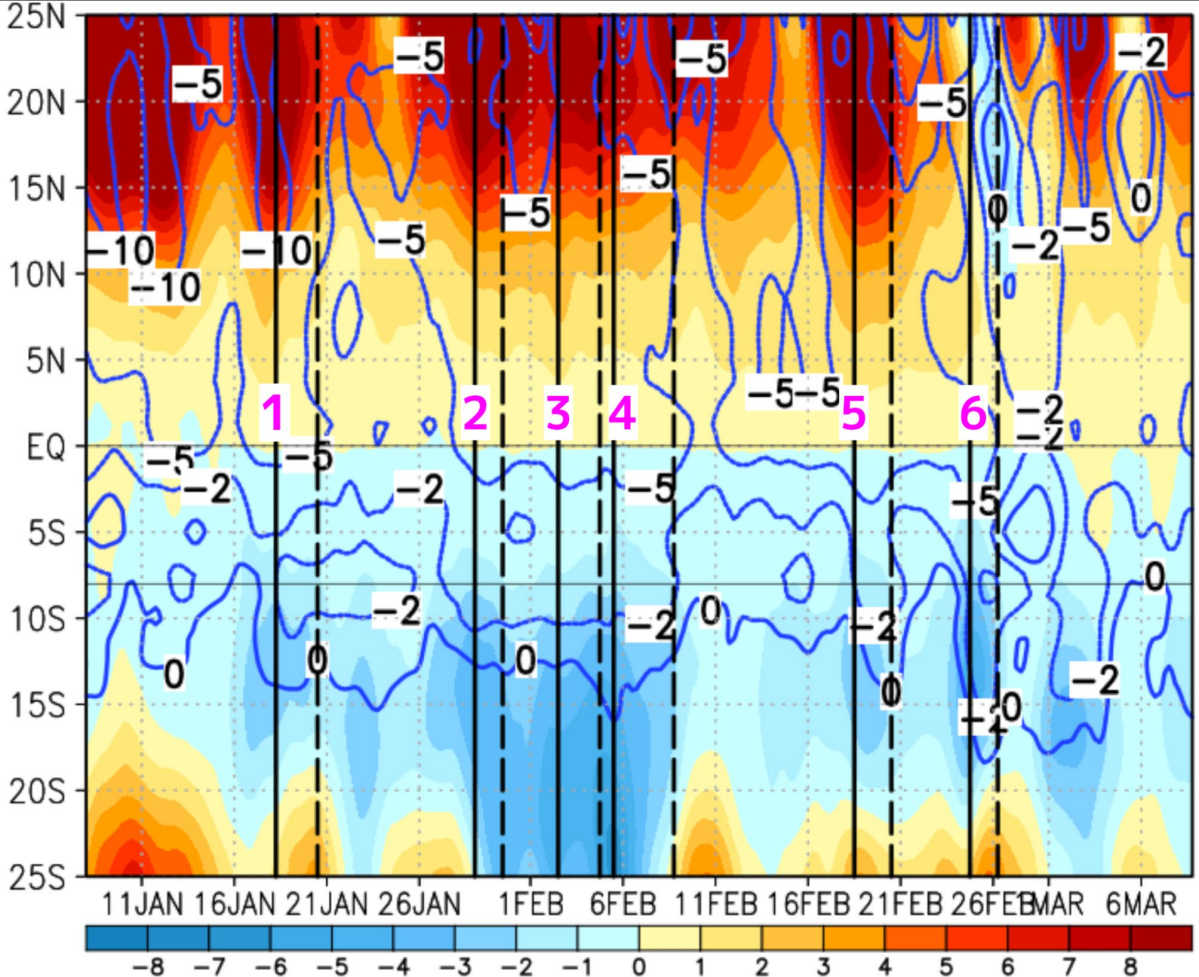
Time–latitude cross-section averaged over 105°E-120°E from January 8 to March 8, 2021. The colors represent the SLP differences relative to the SLP at the equator, and the blue contours indicate the northerly 10-m wind velocity at −10, −5, and −2 m/s. The solid and dashed lines represent the onset and end dates of the 6 CENS events, respectively. The purple numbers denote the CENS events: CENS1, CENS2, CENS3, CENS4, CENS5, and CENS6. The plot was generated with GrADS v2.2.1 (http://cola.gmu.edu/grads/grads.php).

To intensify northerly winds, it is essential to strengthen the southward pressure gradient across the equator. Figure 4 shows the time–latitude cross-section of the SLP differences referenced to the SLP at the equator, which is used to examine the meridional SLP gradient pattern, and northerly winds averaged over 105–120°E during the YMC-CSO2021. Focusing on the SLP differences to the north of 10°N, during the periods of CENS1, CENS2, CENS4, and CENS5, which were associated with the CS, there were significant positive SLP differences of more than 2 hPa. However, in CENS4, the positive SLP difference decayed northward, and in CENS6, it became a negative SLP difference in association with a significant twin-cyclone, which propagated westward from the MJO over the Pacific, after the high-pressure system moving to the east of South China Sea (see Fig. S3f). This fact indicates that the CENS did not simply occur as the southern edge of the southward-expanded CS. Focusing on the SLP differences to the south of 10°S, significant negative SLP differences corresponded to all 6 CENS events (see Fig. S3). In CENS4 and CENS6, which were not associated with the CS, the negative SLP differences significantly deepened to −2 to −3 hPa at 10°S, and the northerly wind expanded southward beyond 15°S. This common feature of significant negative SLP differences corresponding to all 6 CENS events implies that a southward pressure gradient near the low-SLP area between 15°S and 10°S was necessary to enhance the significant northerly winds of the CENS.

### Statistical features of past CENS events

**Fig. 5:**
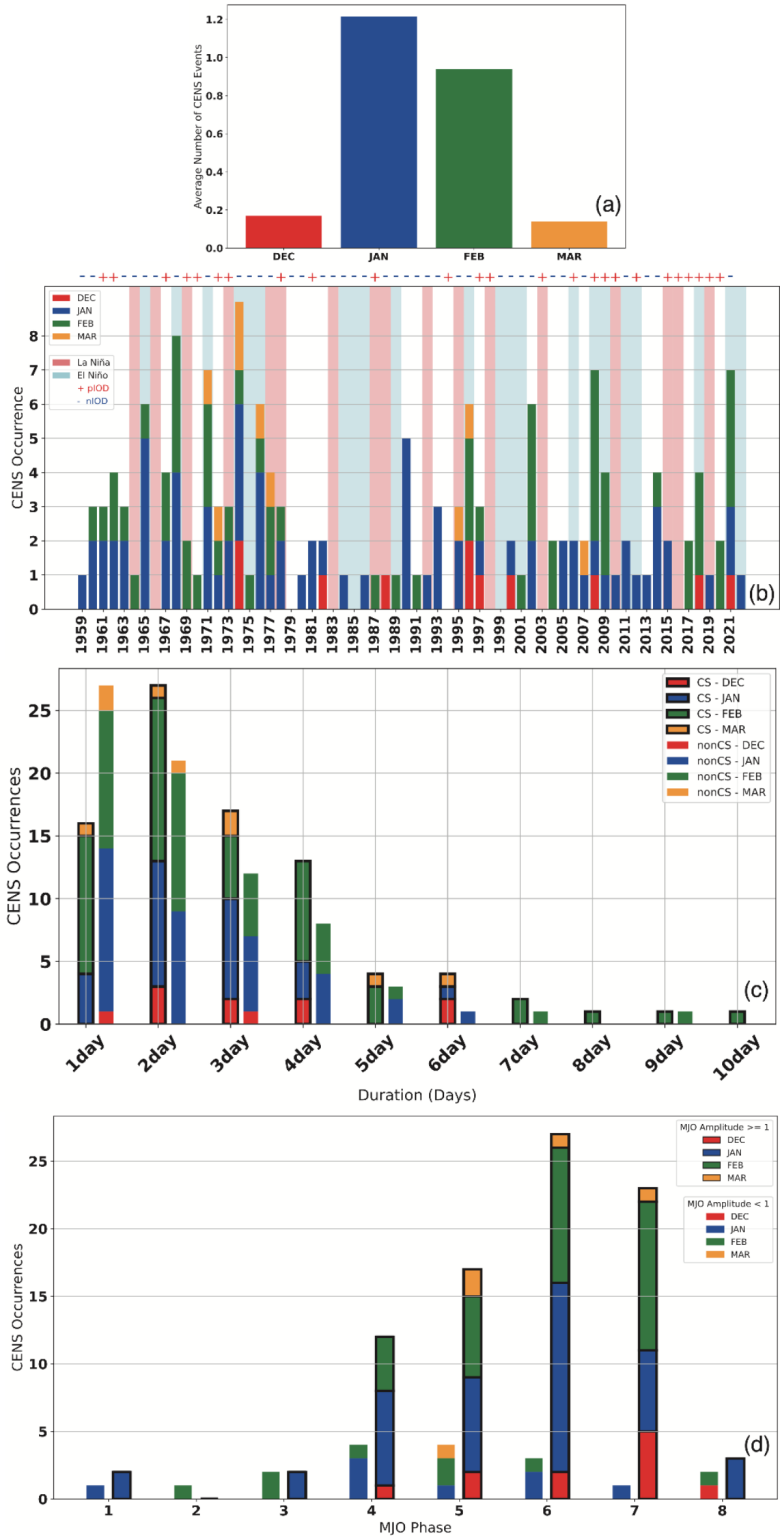
(**a**) Monthly occurrence frequency of CENS events for December, January, February, and March, based on data from 1959 to 2022. (**b**) Time series of CENS occurrences from 1959 to 2022. The El Niño and La Niña periods are represented by pink and light blue backgrounds, respectively. Positive and negative IOD phases are indicated by plus and minus signs at the top of the panel. (**c**) CENS occurrences categorized by duration, with CENS events associated with a CS shown on the left side and those without a CS shown on the right side of each bin. (d) CENS occurrences categorized by the MJO phase, based on data from 1974 to 2022. CENS events with MJO amplitudes less than 1 are shown on the left side, and those with amplitudes of 1 or greater are shown on the right side of each bin. The red, blue, green, and orange bars in all panels represent CENS occurrences in December, January, February, and March, respectively. All plots were generated using Python 3.9.6 (http://www.python.org) including matplotlib 3.8.4.

In this section, to investigate the representativeness of the 6 CENS events observed during the YMC-CSO2021, the statistical characteristics of CENS occurrences and the vertical structure from composite analysis are described. The pressure anomaly fields around the MC significantly fluctuate due to the El Niño-Southern Oscillation (ENSO)^14^, and it is known that the ascending region of the Walker circulation is enhanced during La Niña years^15^. Table 2 summarizes the CENS occurrences by ENSO phase and month for the 160 CENS events, which were extracted from 64 seasons—December, January, February, and March 1959–2022. Over 80% of the 160 CENS events occurred in January and February (Fig. 5a), showing a clear relationship with the ENSO phase (Fig. 5b). That is, the number of CENS occurrences was the lowest during El Niño years and the greatest during La Niña years across all months. However, there was no significant difference in the number of CS and non-CS CENS occurrences in any ENSO phase or monthly classification.

**Table 2:** Counts of CENS occurrences categorized by ENSO phase and month. Each count is divided into CENS events with and without a CS. The ENSO phases, El Niño (E), La Niña (L), and neutral (N) events, were determined based on the NINO 3.4 index averaged for 4 months, December, January, February, and March.

Month	E (17 years)	L (22 years)	*N* (25 years)	Total (64 years)
December	[1, 0]	[8, 0]	[0, 2]	[9, 2]
January	[6, 6]	[20, 13]	[19, 15]	[45, 34]
February	[4, 4]	[14, 17]	[8, 14]	[26, 35]
March	[2, 0]	[3, 2]	[1, 1]	[6, 3]
Total	[13, 10]	[45, 32]	[28, 32]	[86, 74]

As shown in Figs. 5b and 2021 was a La Niña year, with 7 CENS occurrences over 4 months, making it the 3rd highest in the past 64 years. After extracting the 3 years with the greatest number of CENS occurrences, we found 9 in 1974, 8 in 1967, and 7 each in 1970, 2008, and 2021—all of which were La Niña years. During La Niña years, large-scale environmental conditions in the MC region are characterized by lower pressure and enhanced ascending motion in the Walker circulation^14,15^. These conditions could strengthen the southward pressure gradient between the MC and the high-pressure system over the Eurasian continent, making it favorable for intensifying northerly winds compared to El Niño years. The number of CENS occurrences in relation to the positive/negative DMI was 80/80, and there was no significant difference in the number of occurrences when the DMI thresholds were set at ± 0.1, ± 0.2, and ± 0.3. Since the Indian Ocean Dipole (IOD)^16^ is a phenomenon with a larger amplitude from August to November, the impact of the IOD on the occurrence of CENSs could be small.

As shown in Fig. 5c, the duration of most CENSs ranged from 1 to 4 days, and there was no significant difference in the duration of CENSs with or without a CS. As indicated in Table 2, the highest number of CENS occurrences occurred in January, but all long-lived CENS events (lasting from 7 to 10 days) occurred in February. Compared to these statistical characteristics, the durations of the 6 CENS events observed during the YMC-CSO2021 were 3, 2, 3, 4, 2, and 2 days, which can be considered typical durations.

Figure 5d shows the occurrences of 102 CENS events classified by the phase and amplitude of the MJO over a 48-year period from December 1974 to March 2022. First, it is evident that the CENS occurrences were significantly greater during periods when the MJO was active (amplitude of 1 or higher) than during periods when the MJO was inactive (amplitude less than 1). Examining the relationship between the CENS occurrences and the MJO phase, 35% (36 events) of the events occurred in Phases 4 and 5 when the MJO was positioned over the MC, and 52% (53 events) of the events occurred in Phases 6 and 7 when the MJO was positioned over the Pacific Ocean. As shown in Table 1, the 6 CENS events observed during the YMC-CSO2021 occurred 4 times when the MJO was active and 2 times when it was inactive, with 3 CENS events in MJO Phase 6, 2 CENS events in MJO Phase 7, and 1 CENS event in MJO Phase 4, which is consistent with the statistical characteristics indicated by the past 102 events. The fact that 87% (89 out of 102 events) of CENS occurrences took place during MJO Phases 4–7 can be attributed to the low-pressure conditions over the MC associated with the MJO, which could strengthen the southward pressure gradient and produce favorable conditions for intensifying northerly winds.

**Fig. 6:**
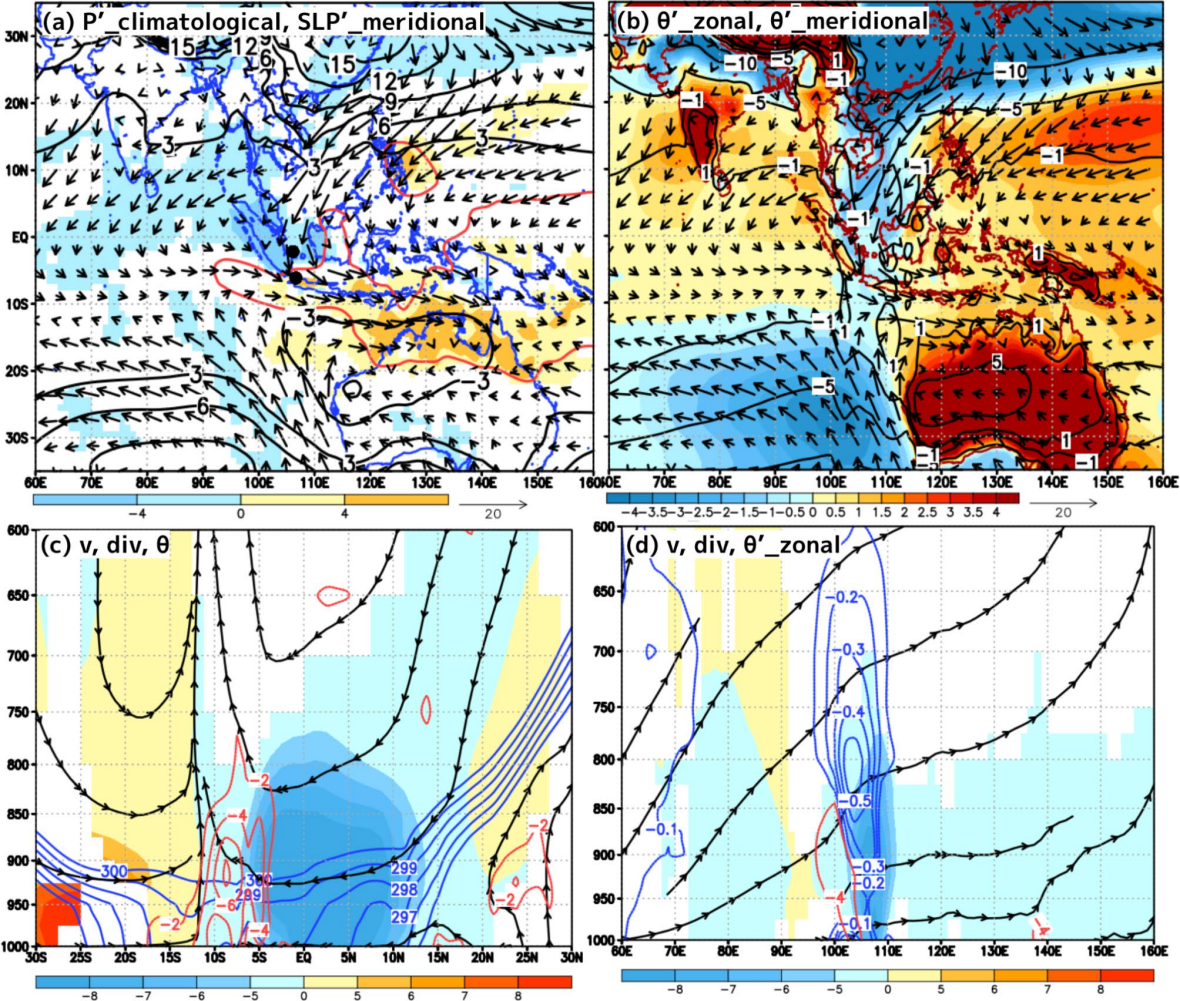
Composite analysis of SLP, PT, winds with JRA-55, and precipitation with GPCP. (**a**) Horizontal distribution of the GPCP precipitation anomaly relative to the climatological mean for December, January, February, and March at the 95% confidence level (colors, mm/day). The red contour represents 10 mm/day of GPCP precipitation. The black contours represent SLP differences relative to the SLP at the equator (every 3 hPa). (**b**) Horizontal distribution of PT differences relative to the PT at 60°E (colors, K) and −5, −1, 1, 5, and 10 K of PT differences relative to the PT at the equator (black contours). (**c**) Latitudinal cross-section averaged from 105–110°E, showing meridional wind velocity (colors, m/s), divergence (red contours at −2, −4, and −6 × 10^−6^ s^[-1]^), and PT (blue contours at 295–300 K, every 1 K). (**d**) Longitude vertical cross-section averaged from 8°S to the equator, showing meridional wind velocity (colors, m/s), divergence (red contours at -4 × 10^−6^ s^[-1]^), and negative PT anomalies (blue contours, every 0.1 K) relative to the PT averaged from 80 to 90°E. The meridional wind velocity in (**c**) and (**d**) is color-shaded to indicate areas that are significantly different from the climatological mean for December, January, February, and March, at the 95% confidence level. The streamlines in (**c**) and (**d**) are drawn by the horizontal wind and vertical pressure velocities. A scale factor of 100 was applied to the vertical pressure velocity. All plots were generated with GrADS v2.2.1 (http://cola.gmu.edu/grads/grads.php).

Figure 6 shows the statistical composited 3-dimensional structure of the past CENS events. Here, we performed a composite analysis over the entire periods that met the CENS criteria, based on 6-hourly JRA-55 reanalysis data. Examining the distribution of the SLP differences referenced to the SLP at the equator shown in Fig. 6a, the surface northerly winds flowing across the MC were noticeable between the positive SLP differences of 9–15 hPa over southeastern Eurasia and the negative SLP differences of −3 hPa over northwestern Australia. In particular, the negative SLP differences below −3 hPa were distributed in the eastern Indian Ocean to the south of the Java and Sumatra Islands, and this feature is consistent with the fact that all 6 CENS events shown in Fig. 4 corresponded with the decreasing SLP in the Southern Hemisphere. A large precipitation area of 10 mm/day (indicated by the red contour), which corresponds to significant positive precipitation anomalies, was distributed ahead of the northerly winds of the CENS (southeastern Sumatra, Java, and southwestern Borneo Islands).

Examining the PT differences at the surface shown in Fig. 6b, the PT over the southern South China Sea, Karimata Strait, and Java Sea were relatively 0.5–1 K cooler than the PT over the Indian Ocean and Pacific Ocean. In other words, there was a locally significant zonal PT gradient crossing Sumatra and the Malay Peninsula at approximately 100°E.

Examining the latitudinal vertical cross-section shown in Fig. 6c, the northerly wind associated with CENS was deepest over the equator (reaching 800 hPa at the 5 m/s threshold) and extended to approximately 5°S. However, the cold air dome (indicated by the 298 K contour) originating from the Eurasian continent became indistinct near the equator. The lower-level convergence at the southern edge of the CENS was located between 15°S and 5°S, which coincided with the region of prominent ascending motion.

Examining the longitudinal vertical cross-section shown in Fig. 6d, in the western part of the northerly wind associated with the CENS (100°E–110°E), there were PT anomalies of −0.1 to −0.5 K (indicated by the blue contours) against the westerly inflow from the Indian Ocean, accompanied by clear lower-level convergence (indicated by the red contour) and stronger ascending motion. These negative PT anomalies are consistent with the radiosonde observations shown in Fig. 3.

## Discussion

Based on the observations and statistical analysis of this study, we discuss two key points associated with the occurrence of CENSs:

(1) Precipitation formation processes around Java Island.

(2) The roles of CENS, MJO, and ENSO in enhancing precipitation in the MC region.

For Issue (1), according to a previous study^4^, CENS events occurring during the active phase of the MJO over the MC (Phases 4–5) enhanced precipitation around Java Island. The positive precipitation anomalies around Java Island are considered to have been due to the low-level convergence between the northerly winds of the CENS and the land‒sea breeze over Java Island, which enhanced the diurnal precipitation cycle over the island^6,10^. While MJO and ENSO influence precipitation patterns on larger spatial and temporal scales, CENS plays a crucial role in enhancing local low-level convergence and precipitation, particularly through its interaction with the land‒sea breeze circulation around Java Island. Although this study did not analyze the diurnal precipitation cycle, the precipitation enhancement around Java Island during the YMC-CSO2021 was also likely driven by the interaction between the CENS and the land‒sea breeze circulation. Furthermore, during CENS events, positive precipitation anomalies associated with a low-pressure anomalies in northwestern Australia were observed over a wide area in the Southern Hemisphere (Fig. 6a). Considering that more than 50% of CENS events coincided with MJO Phases 6–7, these widespread positive precipitation anomalies likely included contributions from the MJO. This study did not separately examine the precipitation enhancement due to the low-level convergence ahead of the southern edge of CENS and that associated with the MJO. Therefore, future work should examine precipitation formation by separating the diurnal component, the enhancement due to CENS, and the MJO component.

For Issue (2), CENS, MJO, and ENSO each contribute to precipitation enhancement, although in distinct ways. ENSO operates on an interannual timescale and influences the basic state over the MC. During La Niña years, large-scale environmental conditions in the MC region are characterized by enhanced ascending motion in the Walker circulation^14,15^, and precipitation over the MC region tend to be enhanced. The MJO, characterized by its eastward propagation, dominantly contribute to precipitation variability over the entire MC. Background zonal circulation patterns over the MC are dominated depending on the MJO phases. These modulations provide a broader-scale context for phenomena such as CENS. In contrast, CENS plays a critical role in modulating local precipitation through the intensification of low-level convergence. This localized impact is achieved through the interaction of cold northerly winds with pre-existing circulations, intensifying low-level ascent and convergence. Even under suppressed MJO phases or during El Niño years, CENS has the potential to enhance rainfall locally, particularly in regions where broader-scale phenomena may not fully account for observed precipitation anomalies. The precipitation enhancement observed during CENS events is not independent of the broader atmospheric systems. Instead, it is the result of multi-scale interactions. For instance, the presence of a low-pressure system over northern Australia, which persists regardless of MJO activity, provides a backdrop for the development of CENS. Similarly, La Niña conditions can amplify the effects of CENS by strengthening the background circulation favorable for CENSs. This study underscores the importance of considering CENS as a distinct driver of local-scale precipitation enhancement, complementing the larger-scale influences of the MJO and ENSO. Each phenomenon operates on a unique spatial and temporal scale, and their combined effects determine the overall precipitation distribution over the MC. Future work should aim to further clarify these interactions, using targeted observational campaigns and modeling.

## Conclusions

This study provides new insights into the vertical structure of CENS events and their associations with CSs, MJO, and ENSO. First, the 6 CENS events observed during the YMC-CSO2021 under different environmental conditions associated with the CS and MJO were investigated in detail. Using radiosonde observations, we identified distinct characteristics in the northerly wind layer thickness, westerly wind bursts, and PT anomalies among the 6 CENS events, confirming the representation of these features by JRA-55. This study highlighted distinct characteristics under different conditions, with CENS1, CENS2, CENS4, and CENS5 linked to the CS and CENS3, CENS4, CENS5, and CENS6 linked to active MJO convection. Notably, CENS6 featured a deep northerly wind layer reaching 400 hPa in association with a significant twin-cyclone to the west of the MJO over the Pacific. In the 64-year statistical analysis, no other cases were detected with similar features to those of CENS6, making it an extremely rare event. The northerly wind thickness of the CENS had distinct features under the different environmental conditions associated with the CS and MJO. On the other hand, the common feature of the CENS was associated with a decrease in SLP between 15°S and 10°S, which produced a southward pressure gradient that enhanced northerly winds.

Statistical analysis of past CENS events revealed higher occurrences during La Niña years and an active MJO located over the western Pacific, consistent with the YMC-CSO2021 observations. Over 80% of the 160 CENS events occurred in January and February, and the number of CENS events was the lowest during El Niño years and the greatest during La Niña years. The duration of most CENS events ranged from 1 to 4 days, and longer-lived CENS events lasting 7 to 10 days tended to occur in February. The durations of the 6 CENS events during the YMC-CSO2021 were all between 2 and 4 days, indicating typical durations. Examining the relationship between the CENS occurrences and the MJO phase, 35% (36 events) of the events occurred in Phases 4 and 5 when the MJO was positioned over the MC, and 52% (53 events) of the events occurred in Phases 6 and 7 when the MJO was positioned over the Pacific Ocean. This statistical trend is also consistent with the 6 CENS events observed during the YMC-CSO2021. Moreover, there was no significant difference in the occurrence or duration of CENSs between the CS and non-CS conditions. Additionally, there was no significant difference in the occurrence of CENSs related to the positive/negative IOD phase.

Composite analysis revealed that the lower-level convergence between the cooler CENS northerly flow and the westerly inflow from the Indian Ocean could enhance significant ascending motion, and precipitation could be significantly intensified along the southern edge of the CENS. The northerly winds of the CENS produced cold air advection from the midlatitudes, extending from the surface to the middle troposphere for 1–10 days, which may act as one of the disruptive factors for the zonal circulation over the MC. Such synoptic-scale cold air advection across the equator could have a feedback or influence on other scale phenomena, such as the diurnal cycle of land/sea breeze circulation and the MJO. Although this study did not directly address the impact of CENSs on the MJO, the cold advection associated with CENSs could contribute to the barrier effects over the MC. The impact of the pronounced negative PT anomalies associated with CENS events on the eastward propagation mechanism and development process of the MJO should be a future issue.

Methods.

We conducted intensive radiosonde observations using the sensors of Meisei iMS-100 from 00 UTC on 8 January to 18 UTC on 8 March 2021 (60 days) at 6-h intervals (00/06/12/18Z) at the Soekarno-Hatta (CGK, 6.12°S, 106.65°E) and Pangkal Pinang (PGK, 2.17°S, 106.13°E) stations, where there is a meteorological station for the Jakarta Soekarno–Hatta International Airport managed by BMKG. All soundings used in this study were averaged vertically into 100-m intervals from the raw data, which had a vertical resolution of approximately 5 m and were recorded at 1-second intervals.

Japanese 55-year reanalysis (JRA-55) data, covering the period from 1958 to the present^17,18^, were used to investigate large-scale atmospheric environmental factors. The dataset had a 1.25° horizontal resolution, 38 levels (including the surface and levels from 1 to 1000 hPa), and 6-h intervals.

The precipitation data from Global Precipitation Climatology Project (GPCP) Version 1.3 at a 1° daily resolution from multisatellite observations, covering the period from October 1996 to the present^19^, were used to analyze the precipitation pattern in association with the CENS.

CENS events were identified using an area average of surface meridional wind velocity from JRA-55, calculated only over the grid points located over the ocean between 105–110°E and 8°S–0°, and subjected to a 24-hour moving average. A threshold of -5 m/s was used to determine the onset, duration, and end of CENS events (see Fig. S1). The cold surge was defined as an event in which the average 925-hPa northerly wind from JRA-55 between 110°E and 117.5°E and along 15°N exceeded 8 m/s^3^. While the cold surge is generally associated with lower temperatures and higher pressure, it is evident that strong northerly winds over the South China Sea during winter are linked to cold air outbreaks from the Eurasian continent. Thus, this study adopts a simplified definition based solely on wind speed.

The Niño 3.4 index was obtained from the Global Climate Observing System Working Group on Surface Pressure at https://www.esrl.noaa.gov/psd/gcos_wgsp/Timeseries/Nino34/.

on 14/10/2024. The Niño 3.4 index typically uses a 5-month running mean, and El Niño or La Niña events are defined when the Niño 3.4 SSTs exceed +/- 0.4 °C or +/- 0.5 °C for a period of six months or more. In this study, El Niño or La Niña features were defined when Niño 3.4 SSTs exceed +/- 0.5 °C for a period of four months (December, January, February, and March) to analyze the trend of CENS occurrences.

The dipole mode index (DMI) for IOD was also obtained from the National Oceanic and Atmospheric Administration (NOAA) climate indices archive https://psl.noaa.gov/gcos_wgsp/Timeseries/DMI/.

on 14/10/2024. DMI measures the strength of the IOD represented by the anomalous SST difference between the western equatorial Indian Ocean (50°E–70°E and 10°S–10°N) and the southeastern equatorial Indian Ocean (90°E–110°E and 10°S–0°). In this study, positive and negative IOD features were defined when the DMI averaged for a period of four months (December, January, February, and March) was positive and negative to analyze the trend of CENS occurrences.

The MJO index^13^ was obtained from the Australian Bureau of Meteorology https://www.bom.gov.au/climate/mjo/ on 14/10/2024. This index divides the location of the MJO into eight phases using equatorially averaged outgoing longwave radiation and the zonal wind fields at 200- and 850-hPa over an area of 15°S–15°N. Each phase represents an approximate geographic location on the globe. Specifically, Phases 8 and 1 correspond to the Western Hemisphere and Africa, respectively, Phases 2 and 3 correspond to the Western and Eastern Indian Ocean, respectively, Phases 4 and 5 correspond to the Western and Eastern MC, respectively, and Phases 6 and 7 correspond to the Western and Central Pacific, respectively (see Fig. S2). The strength of the MJO was approximated by its amplitude, and an amplitude less than 1 was usually considered to be weak, not coherent, or inactive.

Over a 64-year period from December 1958 to March 2022 for the JRAs5, 160 CENS events were identified; over a 48-year period from December 1974 to March 2022 for the MJO index, 102 CENS events were identified; and over a 26-year period from December 1996 to March 2022 for the GPCP, 63 CENS events were identified.

## Electronic supplementary material

Below is the link to the electronic supplementary material.

Supplementary Material 1

## Data Availability

The data supporting the conclusions of this article are available upon request with corresponding author. Please contact the corresponding author for data requests.
