# Supplementary material for: Vertical structure and occurrence patterns of the cross-equatorial northerly surge under different ENSO and MJO phases
Source: Sci Rep. 2024 Nov 24;14:29116. doi: 10.1038/s41598-024-80951-9 (PMC11586435; doi:10.1038/s41598-024-80951-9)
Supplement: Supplementary file 1 — Supplementary Material 1 [file 41598_2024_80951_MOESM1_ESM.docx]

# **Supplementary information**

**Vertical Structure and Occurrence Patterns of the Cross-Equatorial Northerly Surge under Different ENSO and MJO Phases**

Qoosaku Moteki

Japan Agency for Marine-Earth Science and Technology (JAMSTEC), 2-15 Natsushima-Cho Yokosuka City, Kanagawa, 237-0061, Japan

**Corresponding author**

Email: moteki@jamstec.go.jp


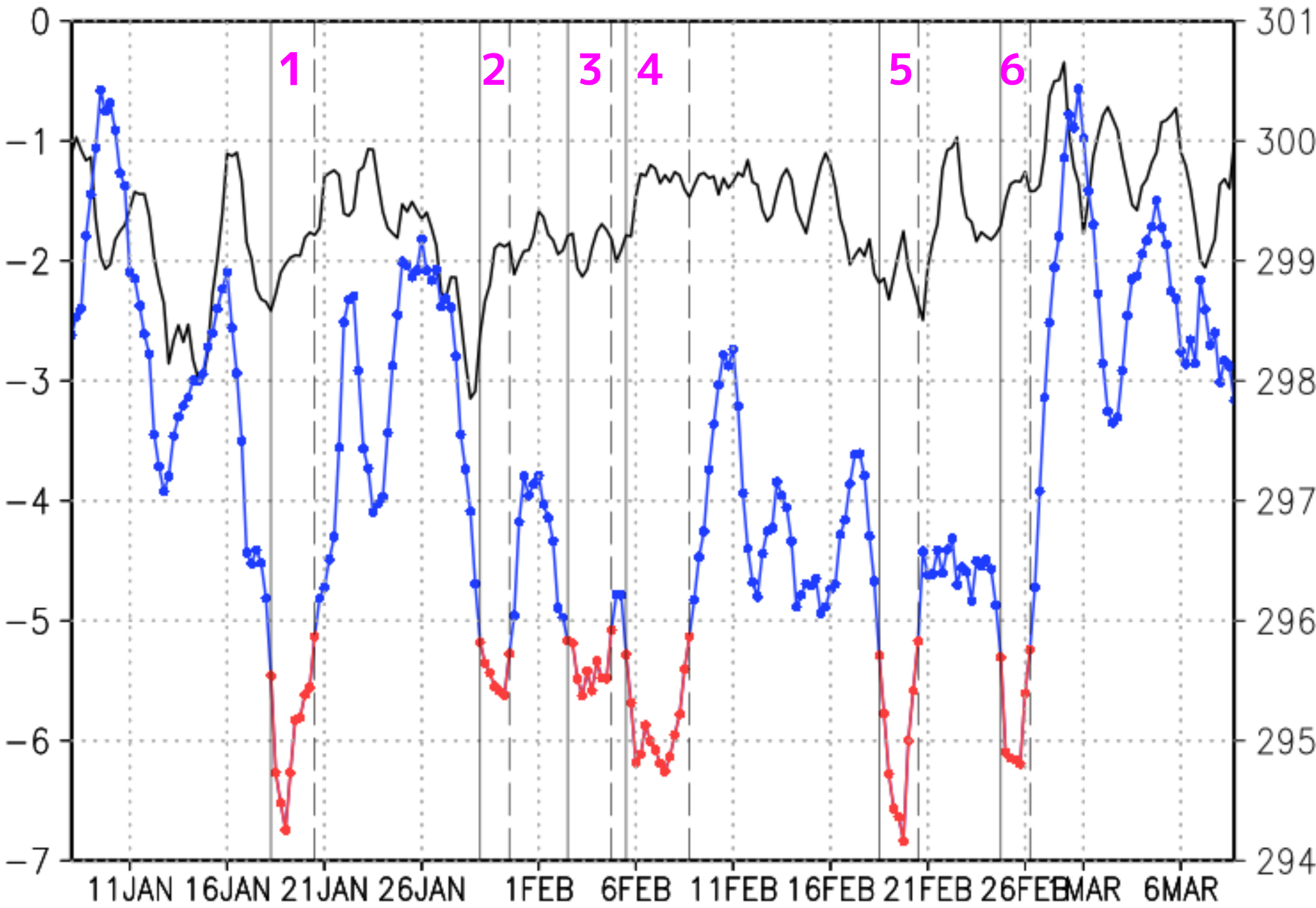


Fig. S1 Time series of the meridional wind velocity (red/blue) and PT (black) from JRA-55 reanalysis averaged over 105°E﹣110°E and 8°S﹣0° from January 8 to March 8, 2021. The red and blue plots represent values below or above the threshold of -5 m/s, respectively. The solid and dashed lines indicate the onset and end dates of the 6 CENS events. The purple numbers denote the CENS events: CENS1, CENS2, CENS3, CENS4, CENS5, and CENS6. The plot was generated with GrADS v2.2.1 (http://cola.gmu.edu/grads/grads.php).


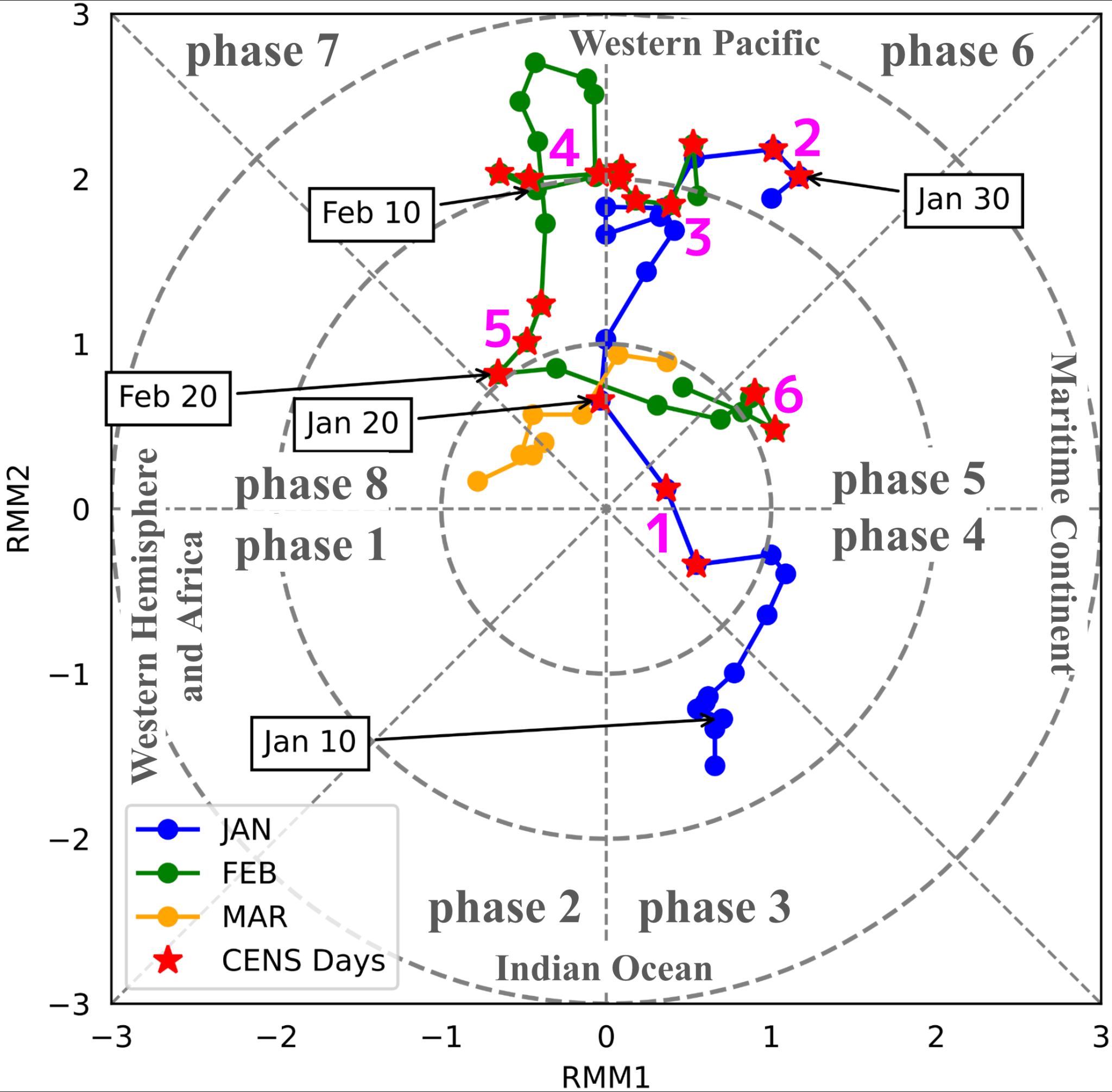


Fig. S2 MJO phase space points for the YMC-CSO2021 period from January 8 to March 8, 2021. Real-time multivariate MJO series 1 and 2 (RMM1, RMM2) were derived from a pair of leading empirical orthogonal functions (EOFs) of MJO variability. The lines and plots colored by blue, green, and orange represent MJO phase space points in January, February, and March, respectively. Star markers indicate the days when CENS occurred. The purple numbers denote the CENS events: CENS1, CENS2, CENS3, CENS4, CENS5, and CENS6. The plot was generated using Python 3.9.6 (http://www.python.org) including matplotlib 3.8.4.

###

###
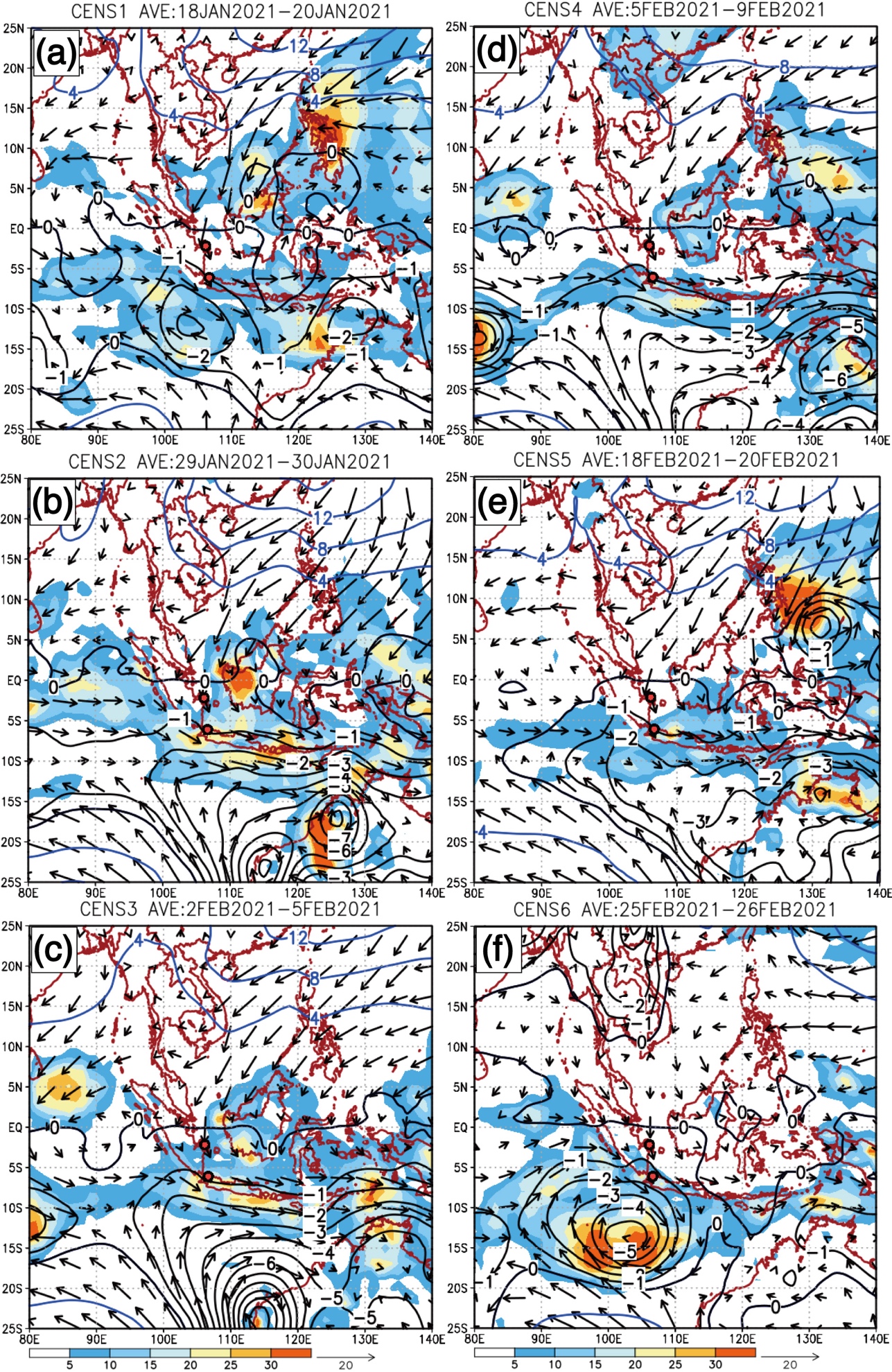


### Fig. S3 Horizontal distribution of precipitation with GPCP (color, mm/day), the SLP differences relative to the SLP at the equator (blue contours for positive and black contours for negative differences, hPa), and 10-m wind vectors from JRA-55 averaged for (a) CENS1 (18-29 Jan), (b) CENS2 (29-30 Jan), (c) CENS3 (2-5 Feb), (d) CENS4 (5-9 Feb), (e) CENS5 (18-20 Feb), and (f) CENS6 (25-26 Feb). The plot was generated with GrADS v2.2.1 (http://cola.gmu.edu/grads/grads.php).
